# Key species drive community and functional stability of segment‐specific gut microbiomes after the swimming crab molting

**DOI:** 10.1002/imo2.51

**Published:** 2025-01-06

**Authors:** Weichuan Lin, Mingming Niu, Changkao Mu, Chunlin Wang, Yangfang Ye

**Affiliations:** ^1^ Marine Economic Research Center, Donghai Academy Ningbo University Ningbo China; ^2^ Key Laboratory of Marine Biotechnology of Zhejiang Province, School of Marine Sciences Ningbo University Ningbo China

## Abstract

Molting is a crucial process for crab growth and development. However, the impacts of molting on the structure and function of the gut bacterial community in swimming crab *Portunus trituberculatus* are poorly understood. Then, dynamic changes in the microbiotas of gut segments (foregut, midgut, and hindgut) after molting were investigated using 16S rRNA gene amplicon and shotgun metagenomic sequencing. We highlight the segment‐specific responses in bacterial community compositions, alpha‐diversity, and co‐occurrence patterns, emphasizing the significant impact of hindgut bacteria on the analysis of the whole gut. The identification of enriched and emerged species and their source, coupled with insights into functional stability and multifunctionality, adds granularity to our understanding of postmolt microbial ecology. We offer potential keys to driving microbial community succession. These findings provide essential insights into the stability and dynamics of gut microbiota, which are crucial for both ecological understanding and sustainable management of crab probiotic regulation.

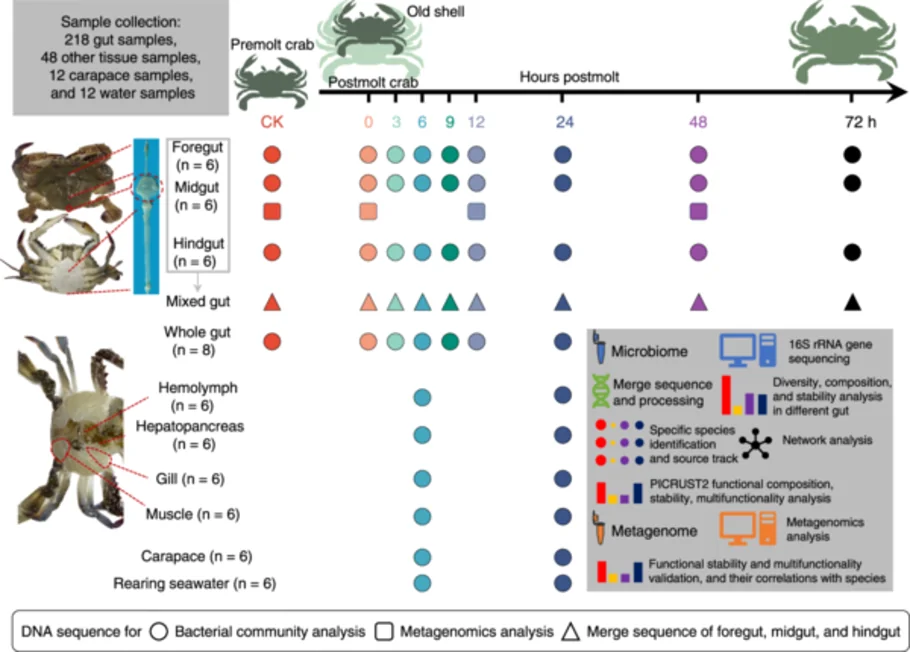

The gut microbiota of animals gradually assembles and matures as the host grows. However, the microbiota of crab is special as it is frequently reassembled due to host molting [1, 2]. Despite the reduction of microbial diversity in carb molting, the community stability may be maintained through species compensation. A narrow group of species may play a larger role in the community structure, function, and stability than the whole community [3, 4]. In this case, it is necessary to reveal species turnover, especially a narrow group of species, during the postmolt recovery of crabs. Spatially, gut segments exhibit functional differences, and segment‐specific microbiota data are needed [5, 6]. However, the changes in the structure and function of segment‐specific gut bacterial communities after crab molting still lack characterization. In this study, we investigated the bacterial community dynamics and differences across gut segments of the swimming crab *Portunus trituberculatus* after molting using 16S rRNA gene amplicon and shotgun metagenomic sequencing. We aim to determine (I) the bacterial community temporal dynamics and differences between gut segments after crab molting; (II) the differential species turnover and the sources of characteristic bacterial clusters in each segment of gut during postmolt; and (III) the role of important taxa in the functional stability and multifunctionality of midgut bacterial community during postmolt and their possible sources.

## 1 RESULTS AND DISCUSSION

To explore the differences in the bacterial community between each gut segment or the whole gut, the samples from foregut, midgut, hindgut, whole gut, other tissues, and carapace surface of crabs as well as rearing water were collected for microbiome analysis (see details in the *Graphical abstract*). Moreover, we merged the sequencing data from each gut segment at each sampling time point into a new single data set by the merge command of USEARCH, thus constructing mock sequencing data of whole gut bacteria (referred to as the mixed gut group).

We found distinct bacterial community compositions in different gut segments compared to the whole gut (Figure S1A). The foregut and midgut had a similar bacterial composition and were dominated by Betaproteobacteria (70.87% and 61.09%, respectively), while the hindgut was dominated by Gammaproteobacteria (60.99%) (Figure S2). Community dissimilarity analysis revealed significant temporal variations in the midgut, hindgut, mixed gut, and whole gut after molting, whereas the foregut showed no such changes (Figure S1B; Figure S3). Alpha‐diversity analysis revealed distinct succession patterns: the richness in the foregut and midgut was decreased at the early stage, whereas the Shannon index of the hindgut was increased at the late stage (Figure S1C,D). The mixed and whole gut exhibited more dramatic changes than individual segments (Figure S1C–E). Linear model analysis indicated the hindgut had the strongest correlation with both the mixed gut and whole gut in terms of α‐diversity and stability (Figure S4).

From the perspective of co‐occurrence patterns, we also found that co‐occurrence patterns varied among gut segments, with fewer changes in the foregut compared to the midgut and hindgut (Figure S5A–C; Figure S6). In particular, node and edge numbers, as well as average degree, decreased in the foregut and midgut but increased in the hindgut after molting (Figure S5A–C; Figure S6A). The average path length decreased significantly in the foregut while it increased in the hindgut (Figure S6B). Clustering coefficients showed little change across all gut segments except at certain time points (Figure S6C). Modularity significantly increased in the midgut (Figure S6D). Network stability followed the average degree trends (Figure S6E), and vulnerability remained relatively stable (Figure S6F). When examining the mixed and whole gut samples, the co‐occurrence patterns and topological characteristics of the mixed gut reflected a mixture of those seen in the three individual gut segments (Figure S5D; Figure S6), while largely differed between the whole gut and each gut segment (Figure S5E; Figure S6). Correlation analysis revealed that only the hindgut network was significantly correlated with the mixed and whole gut networks (*p* < 0.05, Figure S7), confirming that the whole gut microbiome was primarily derived from the hindgut. Thus, if we take the whole gut, the obtained information on the gut bacterial community could be mainly derived from the hindgut bacteria. The different gut segments serve as habitat filters to harbor distinctive microbial community [6, 7], and the midgut is more important as the digestion and absorption take place [8]. Several recent studies have attached importance to the midgut and hindgut bacterial communities of aquatic animals [9, 10]. Therefore, it is necessary to unravel specific characteristics of bacterial communities in the midgut and hindgut for the research of gut bacteria‐host interplay. Notably, our study captures the fine‐scale succession pattern of each gut segment after the swimming crabs newly molt, which complements the previous findings in which only changes of midgut bacterial community have been revealed after 3 d postmolt [2].

To explore the development patterns of gut microbiota due to crab molting, two types of amplicon sequence variants (ASVs) were identified in each group after being compared to those at 0 h postmolt: emerged and enriched. For the CK group, the two kinds were the lost and depleted ASVs, respectively. In the foregut, 592 ASVs (3.35%, total relative abundance) were lost after molting (Figure S8A), and similar losses occurred in the midgut (900 ASVs, 5.04%) and hindgut (520 ASVs, 1.79%) (Figure S8B, C). Likewise, depleted ASVs included four (3.31%, foregut, Figure S8D,G), 40 (4.76%, midgut, Figure S8E,H), and 32 (11.27%, hindgut, Figure S8F,I). More emerged and enriched ASVs were observed in the hindgut than in the foregut and midgut (Figure S8). The hindgut had the most emerged ASVs during postmolt, peaking at 24 h (782 ASVs), and the relative abundance of these ASVs was higher than 10% during this period. Notably, some newly emerged ASVs in the hindgut (e.g., Firmicutes and Deltaproteobacteria) were absent during premolt, while foregut and midgut ASVs were similar to those lost during premolt. Regarding enriched ASVs, the foregut, midgut, and hindgut displayed varying quantities at different postmolt times, with the hindgut showing significant enrichment. The highest total relative abundance of enriched ASVs occurred at 72 h postmolt for the midgut (26.68%) and hindgut (22%) (Figure S8).

Next, we conducted the tipping point analysis to determine whether and when the gut bacterial communities tended to be stable again. We found that there was no significant change in the foregut bacterial community throughout the postmolt period (Figure S3A, Figure S9A). However, the tipping points were found in the midgut (24 h) and hindgut (21.3 h) (Figure S9B,C), which suggests that the bacterial communities of the midgut and hindgut tended to be stable after 24 h postmolt. The recovery of midgut and hindgut bacterial communities may attribute to the proliferation of indigenous bacteria and the immigration of exotic bacteria. Source tracking analysis offered some support for this view. It showed that the bacteria of the carapace surface and seawater significantly contributed to the newly emerged ASVs in all gut segments, although a large proportion of sources remained unknown at 48 h postmolt (Figure S10A–C). For the enriched ASVs, the bacteria from the same gut segments at 24 h were the primary sources for the foregut and midgut (Figure S10D,E), while carapace surface bacteria (83.79%) contributed the most to the hindgut at 48 h postmolt (Figure S10F). Generally, exotic bacteria are hard to colonize in the gut [11]. Thus, it seems to be reasonable that indigenous microbes make a greater contribution to the recovery of the midgut bacterial community than exotic bacteria. However, this is not the case in the hindgut. The morphology of the hindgut, characterized by its direct linkage with the carapace surface and opening to the outside environment, probably explains the reason of the major source from the carapace surface.

**FIGURE 1:**
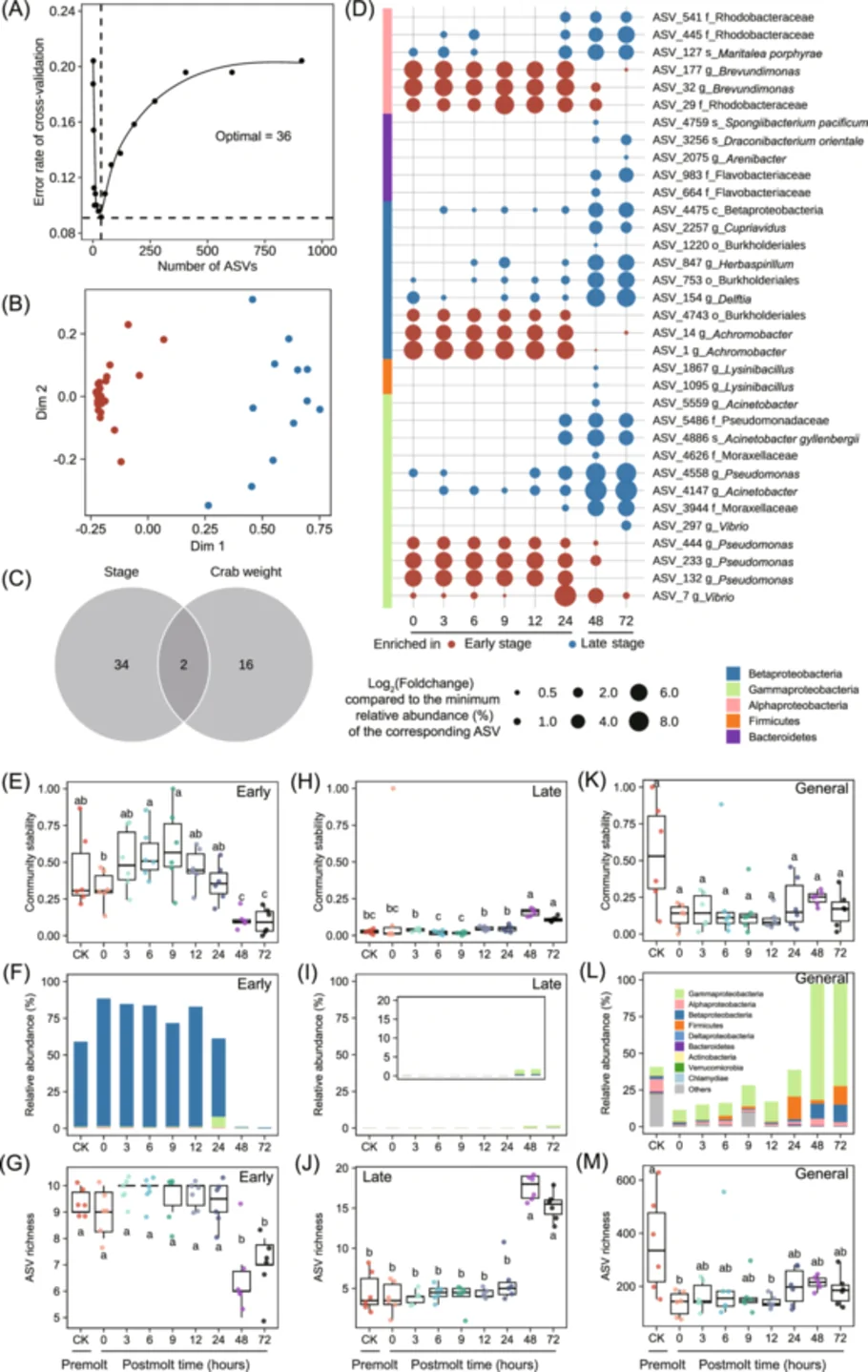
Bacterial taxa of midgut that enriched at the early and late stages during postmolt. (A) Tenfold cross‐validation error as a function of the number of input bacterial taxa used to differentiate early and late stages in order of variable importance. (B) Nonmetric multidimensional scaling (NMDS) plot based on Bray–Curtis dissimilarity visualizing compositional variations of 36 bacterial taxa at the early (red) and late (blue) stages. (C) Venn plot depicting the intersection of bacterial taxa related to stages and crab weight. (D) Changes in the relative abundances of 34 bacterial taxa at the early and late stages. The size of each point represents the fold change compared to the minimum value within each bacterial taxon. The color of the point represents each bacterial taxon enriched at the early (red) or late (blue) stage. The colors of the bar on the left mean different phyla. Community stability, relative abundances, and ASV richness of early (E–G), late (H–J), and general ASVs (K–M) of midgut bacteria. Early and late ASVs serve as indicators of postmolt early and late stages, respectively. General ASVs represent species that lack distinctiveness in differentiating between the early and late stages. Different lowercase letters indicate significant differences between groups (*p* < 0.05), as determined by Kruskal–Wallis test and Benjamini & Hochberg *p*‐value correction.

To investigate drivers and their roles in community stability and diversity during postmolt, a 24‐h mark was first determined based on the tipping points to divide the early (0–24 h) and late (48–72 h) stages and the random forest classification (RFC) model was then constructed to identify key taxa for each stage. In the midgut, 34 out of 36 ASVs showed distinct differences between the early and late stages (Figure 1A–C). Among them, 10 ASVs were enriched at the early stage, which mainly belonged to *Brevundimonas*, *Achromobacter*, and *Pseudomonas* (Figure 1D). The rest (24 ASVs) were enriched at the late stage, including Rhodobacteraceae, Flavobacteriaceae, *Lysinibacillus*, and *Acinetobacter*. When doing the same RFC model construction for the hindgut bacteria, the top 19 ASVs were found (Figure S11A,B) and no ASVs were closely linked to crab weight (Figure S11C). Of them, five ASVs and 14 ASVs were enriched at the early and late stages, respectively (Figure S11D).

We then divided the bacterial communities in the midgut and hindgut into three clusters: early, late, and general. In the midgut, early ASVs had higher stability, relative abundance, and richness at the early stage (Figure 1E–G), while late ASVs exhibited similar trends at the late stage (Figure 1H–J). The rest ASVs (general ASVs) showed no significant change in community stability and ASV richness, although their total relative abundances were greater at the late stage than at the early stage (Figure 1K–M). These findings implicate that the specific bacteria work in the midgut at a given postmolt stage. Such a species turnover could maintain the stability of the bacterial community [12]. Moreover, late ASVs with less than 2% relative abundance contributed more to stability and richness than general ASVs with over 90% (Figure 1E–M). In contrast, only late ASVs (with 0.12%–7.11% relative abundance) in the hindgut showed consistent improvements in stability and richness throughout postmolt (Figure S12). These results suggest the important role of rare species in improving the community stability [13] and indicate that richness is more important for the community stability than species abundance [14].

**FIGURE 2:**
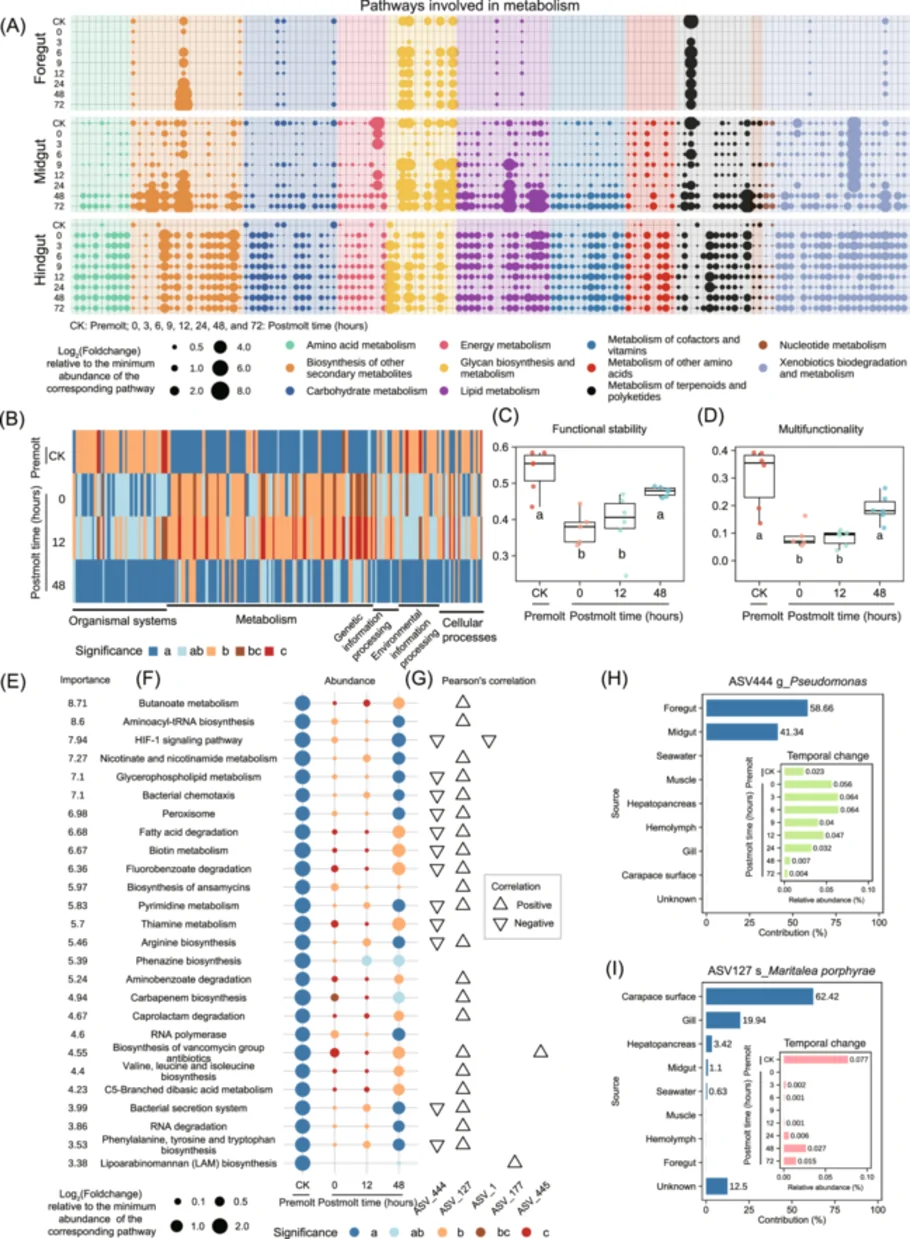
Changes in the metabolic functions of midgut bacteria after molting. (A) Predicted metabolic functions in the foregut, midgut, and hindgut based on PICRUSt2 analysis. The size of each point represents the fold change compared to the minimum value within each pathway. The colors of the point represent different categories of metabolism. (B) Functional succession of midgut bacteria by metagenomics. The colors of the point represent the difference in each function between groups. (C, D) Functional stability and multifunctionality of midgut bacteria. (E) Importance ranking of main functions related to functional stability and multifunctionality. (F) Changes in the abundance of key functions. The size of each point represents the fold change compared to the minimum value within each bacterial taxon. The colors of the point represent the difference in each function between groups. (G) Correlation between main functions and enriched bacterial taxa. Only bacterial taxon significantly correlated with functions was visualized. The shapes mean significantly positive (upper triangle) and negative (lower triangle) correction. (H, I) The potential sources of ASV 444 at 9 h postmolt and ASV 127 at 48 h postmolt, with insets showing relative abundances of these two ASVs over time. Different lowercase letters indicate significant differences between groups (*p* < 0.05), as determined by the Kruskal–Wallis test and Benjamini & Hochberg *p*‐value correction.

To explore the changes in microbial function due to crab molting, we first predicted the function potentials of each gut segment by the PICRUSt2 pipeline. A more drastic shift in the relative abundances of function potentials was observed in the midgut and hindgut than in the foregut (Figure 2A; Figure S13A). The relative abundances of 119 and 120 metabolic pathways were significantly changed in the midgut and hindgut bacteria, respectively, including amino acid, carbohydrate, and energy metabolism (*p* < 0.05, Figure 2A). However, only in the midgut, did we observe the enhanced differences in bacterial functional compositions with the increasing time intervals (*R* ^2^ = 0.082, *p* < 0.05; Figure S13B). Thus, the typical midgut samples at premolt, as well as at 0, 12, and 48 h postmolt were used in further metagenomic sequencing analysis. The metagenomic sequencing indicated that 271 functional pathways in the midgut (59.5% of the total) were significantly affected (Figure 2B). Pathways related to organismal systems, genetic information processing, and cellular processes increased, while metabolism pathways recovered to premolt level. Functional stability and multifunctionality of the midgut decreased significantly until 48 h postmolt (Figure 2C,D). The RFC model identified 26 functional pathways, including metabolic, genetic information processing, cellular processes, and environmental information processing, as key to functional stability changes (*p* < 0.05, Figure 2E). At 48 h postmolt, 15 pathways, such as aminoacyl‐tRNA biosynthesis, HIF‐1 signaling pathway, and nicotinate and nicotinamide metabolism, recovered to premolt levels (Figure 2F). Further, we found that ASV 444 (*Pseudomonas*) had significant and negative correlations with 12 functional pathways whereas ASV 127 (*Maritalea porphyrae*) had significant and positive correlations with 21 functional pathways (Figure 2G), indicating the vital roles of these two ASVs in the recovery of functional stability and multifunctionality. ASV 444 was primarily sourced from the foregut (58.66%) and midgut (41.34%) (Figure 2H), while ASV 127 originated from the carapace surface (62.42%), gill (19.94%), and hepatopancreas (19.94%) (Figure 2I).

Both compositional and functional stability are considered as indicators of community recovery [15]. Our study indicates a coupled structure and function of gut bacterial communities, which agrees with some studies that compositional and functional stability are positively associated [16, 17]. Moreover, specific species may largely determine ecosystem functioning [18]. The comprehensive correlations of ASV444 and ASV127 to the metabolic functions of bacterial community in the midgut probably support the previous studies that suggest functional stability is likely influenced by a limited group of species [3, 4]. Especially for ASV127 identified as *Maritalea porphyrae*, it plays a beneficial role in promoting the growth of other bacteria [19]. Furthermore, ASV127 was mainly sourced from the carapace surface. Thus, the utilization of this bacterium via immersing could be valued to increase the function of the crab gut.

## 2 CONCLUSION

In conclusion, we examined the effects of molting on the structure and function of gut bacteria in *P. trituberculatus*. Our findings revealed segment‐specific variations in the diversity and composition of gut bacterial communities after crab molting, with the hindgut bacteria contributing most significantly to the whole gut microbiome. Furthermore, the functions of bacterial communities across the three gut segments were closely linked to their structural changes, with a transient reduction in functional stability and multifunctionality observed in the midgut bacterial community. Notably, ASV444 and ASV127 presented significant correlations with functions of the midgut bacterial community. Taken together, these results highlight the intricate relationship between host behavior and the gut microbiome, providing valuable insights for the research on interactions between crabs and their gut microbiota and underscoring the segment‐specific of gut structure and function.

## AUTHOR CONTRIBUTIONS

**Weichuan Lin**: Conceptualization; methodology; investigation; formal analysis; writing—original draft. **Mingming Niu**: Investigation; writing—review and editing. **Changkao Mu**: Writing—review and editing. **Chunlin Wang**: Writing—review and editing; funding acquisition. **Yangfang Ye**: Conceptualization; writing—review and editing; funding acquisition.

## ETHICS STATEMENT

All experimental procedures and animal care were conducted in accordance with the Animal Research Institute Committee Guidelines for Ningbo University, China, and were approved by the Institutional Animal Care and Use Committee (IACUC) of Ningbo University.

## CONFLICTS OF INTEREST STATEMENT

The authors declare no conflicts of interest.

## Supporting information


**Figure S1:** Bacterial community structure in crab gut segments.
**Figure S2:** The top 10 phyla or proteobacteria classes in different gut segments.
**Figure S3:** Community dissimilarity between groups.
**Figure S4:** Linear relationships of α‐diversity indices and community stability of bacterial communities between each gut segment and mixed gut (A) or whole gut (B).
**Figure S5:** Co‐occurrence networks of bacterial communities in different gut segments.
**Figure S6:** Temporal changes in network topological attributions.
**Figure S7:** Linear relationships of network topological attributions between each gut segment and mixed gut (A) or whole gut (B).
**Figure S8:** Temporal changes in the emerged (A–C) and enriched bacterial taxa (D–I) of different gut segments.
**Figure S9:** Tipping points in the changes of bacterial community dissimilarity.
**Figure S10:** The potential sources of the emerged (A–C) and enriched bacterial taxa (D–F) at 48 h postmolt.
**Figure S11:** Bacterial taxa of the hindgut that enriched at the early and late stages during postmolt.
**Figure S12:** Community stability, relative abundances, and ASV richness of early (A–C), general (D–F), and late ASVs (G–I) of hindgut bacteria.
**Figure S13:** Bacterial function changes in different gut segments over postmolt time.

## Data Availability

The data that support the findings of this study are openly available in Sequence Read Archive at https://www.ncbi.nlm.nih.gov/bioproject/PRJNA995221, reference number PRJNA995221. All the raw data used in this research are available in the NCBI Sequence Read Archive (SRA) database (BioProject: PRJNA995221, at: https://www.ncbi.nlm.nih.gov/bioproject/PRJNA995221). The data and scripts used are saved in GitHub (https://github.com/WeichuanLin6688/molt-crab-gut). Supplementary materials (methods, figures, graphical abstract, slides, videos, Chinese translated version, and update materials) may be found in the online DOI or iMetaOmics (http://www.imeta.science/imetaomics/).
