# Supplementary material for: Key species drive community and functional stability of segment‐specific gut microbiomes after the swimming crab molting
Source: IMetaOmics. 2025 Jan 6;2(1):e51. doi: 10.1002/imo2.51 (PMC12806196; doi:10.1002/imo2.51)
Supplement: Supplementary file 1 — Figure S1: Bacterial community structure in crab gut segments. Figure S2: The top 10 phyla or proteobacteria classes in different gut segments. Figure S3: Community dissimilarity between groups. Figure S4: Linear relationships of α‐diversity indices and community stability of bacterial communities between each gut segment and mixed gut (A) or whole gut (B). Figure S5: Co‐occurrence networks of bacterial communities in different gut segments. Figure S6: Temporal changes in network topological attributions. Figure S7: Linear relationships of network topological attributions between each gut segment and mixed gut (A) or whole gut (B). Figure S8: Temporal changes in the emerged (A–C) and enriched bacterial taxa (D–I) of different gut segments. Figure S9: Tipping points in the changes of bacterial community dissimilarity. Figure S10: The potential sources of the emerged (A–C) and enriched bacterial taxa (D–F) at 48 h postmolt. Figure S11: Bacterial taxa of the hindgut that enriched at the early and late stages during postmolt. Figure S12: Community stability, relative abundances, and ASV richness of early (A–C), general (D–F), and late ASVs (G–I) of hindgut bacteria. Figure S13: Bacterial function changes in different gut segments over postmolt time. [file IMO2-2-e51-s001.docx]

**Supporting information to**

**Key species drive community and functional stability of segment-specific gut microbiomes after the swimming crab molting**

**Running title：Key species drive gut microbiome stability**

Weichuan Lin^1, #^, Mingming Niu^1, #^, Changkao Mu^1,2^, Chunlin Wang^1^, Yangfang Ye^1,*^

*^1^ Marine Economic Research Center, Donghai Academy, Ningbo University, Ningbo, 315832, China*

*^2^ Key Laboratory of Marine Biotechnology of Zhejiang Province, School of Marine Sciences, Ningbo University, Ningbo, 315832, China*

#These authors contributed equally: Weichuan Lin, Mingming Niu

*Correspondence: [yeyangfang@nbu.edu.cn](mailto:yeyangfang@nbu.edu.cn) (Yang-fang Ye)

Ningbo University, Ningbo, 315211, China


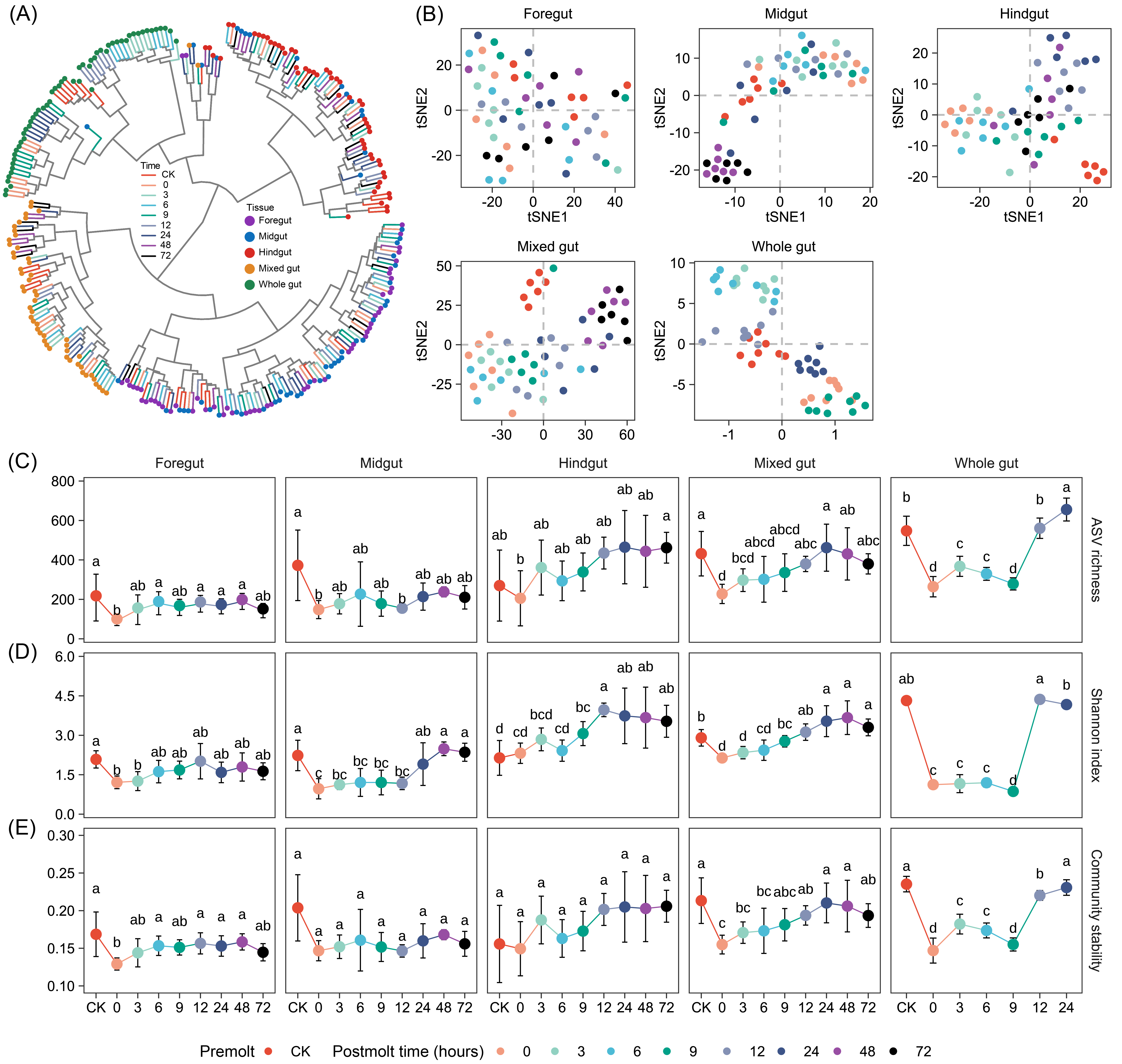


**Figure S1** Bacterial community structure in crab gut segments. (A) Clustering dendrogram using Bray-Curtis as a metric to compare the similarity of bacterial communities between gut segments. Branche and point colors indicate the different time and gut segments, respectively. (B) T-distribution stochastic neighbor embedding (t-SNE) plot visualizing compositional variations of bacterial communities. (C–E) The ASV richness, Shannon index, and stability of bacterial community between gut segments. Different lowercase letters indicate significant differences between groups (*p* < 0.05), as determined by Kruskal-Wallis test and Benjamini & Hochberg *p*-value correction.


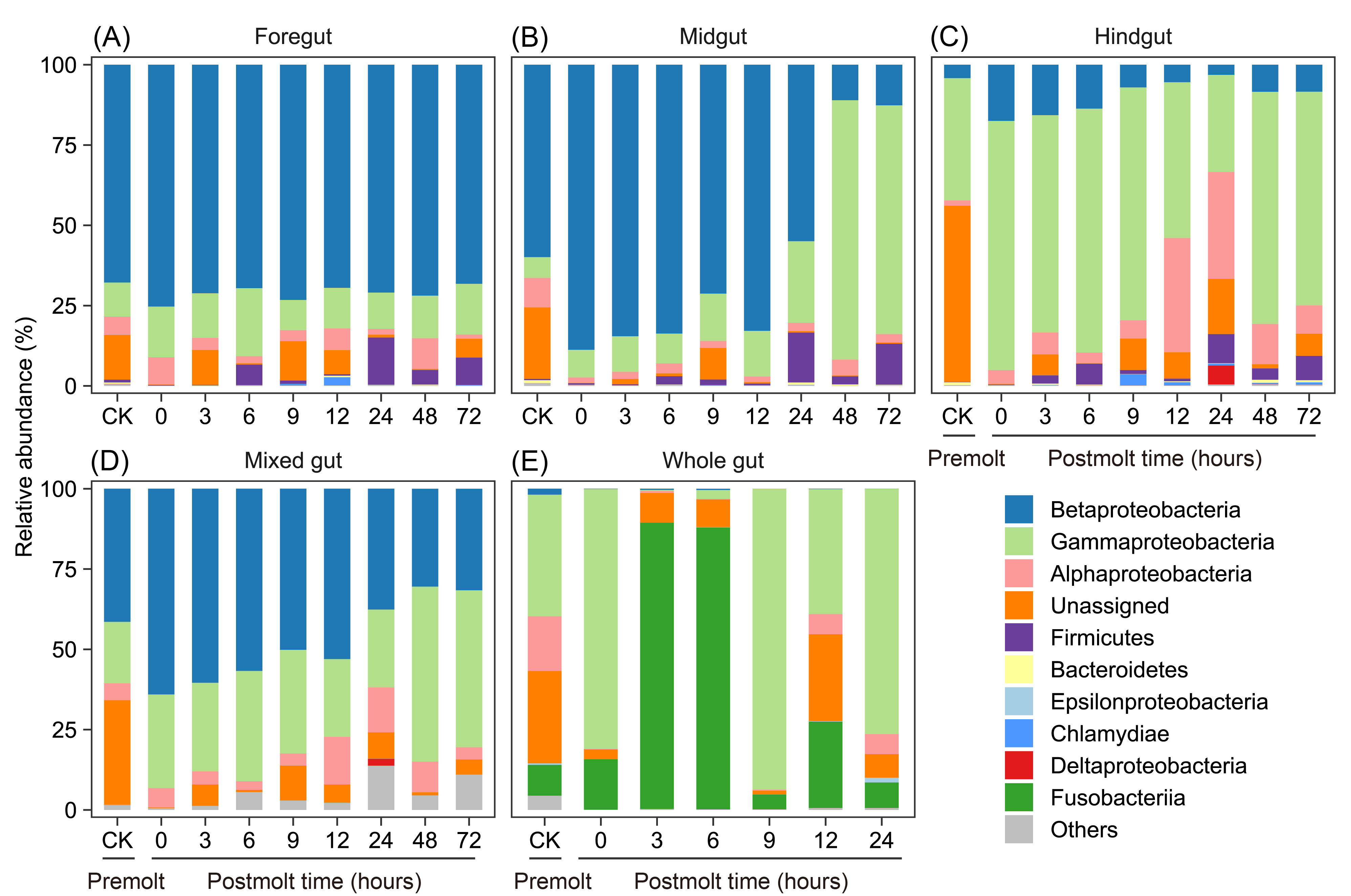


**Figure S2** The top 10 phyla or proteobacteria classes in different gut segments. (A) Foregut. (B) Midgut. (C) Hindgut. (D) Mixed gut. (E) Whole gut.


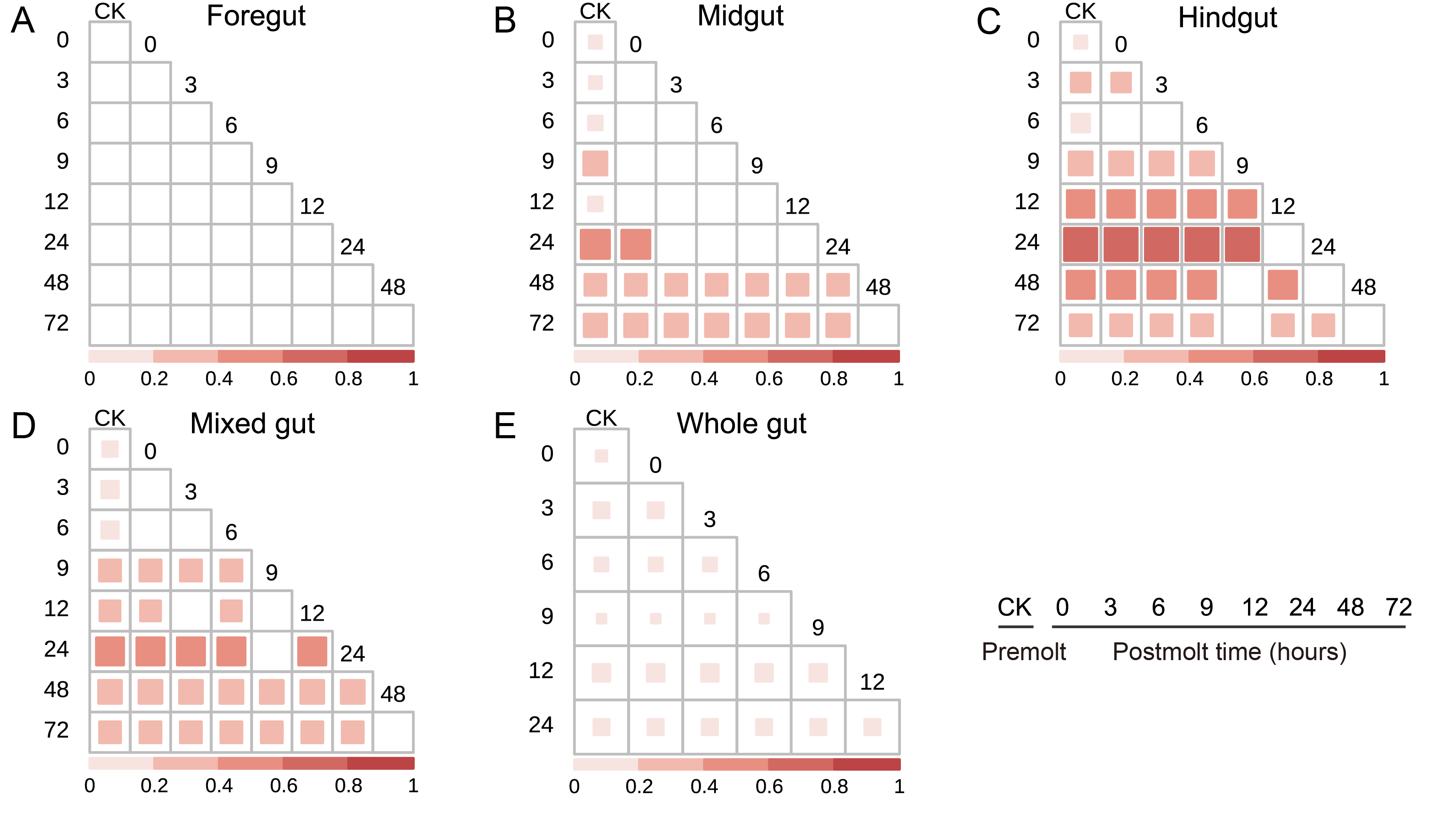


**Figure S3** Community dissimilarity between groups. (A) Foregut. (B) Midgut. (C) Hindgut. (D) Mixed gut. (E) Whole gut. The size and color of the points are proportional to the degree of dissimilarity. Only significant dissimilarities between groups are shown (*p* < 0.05).


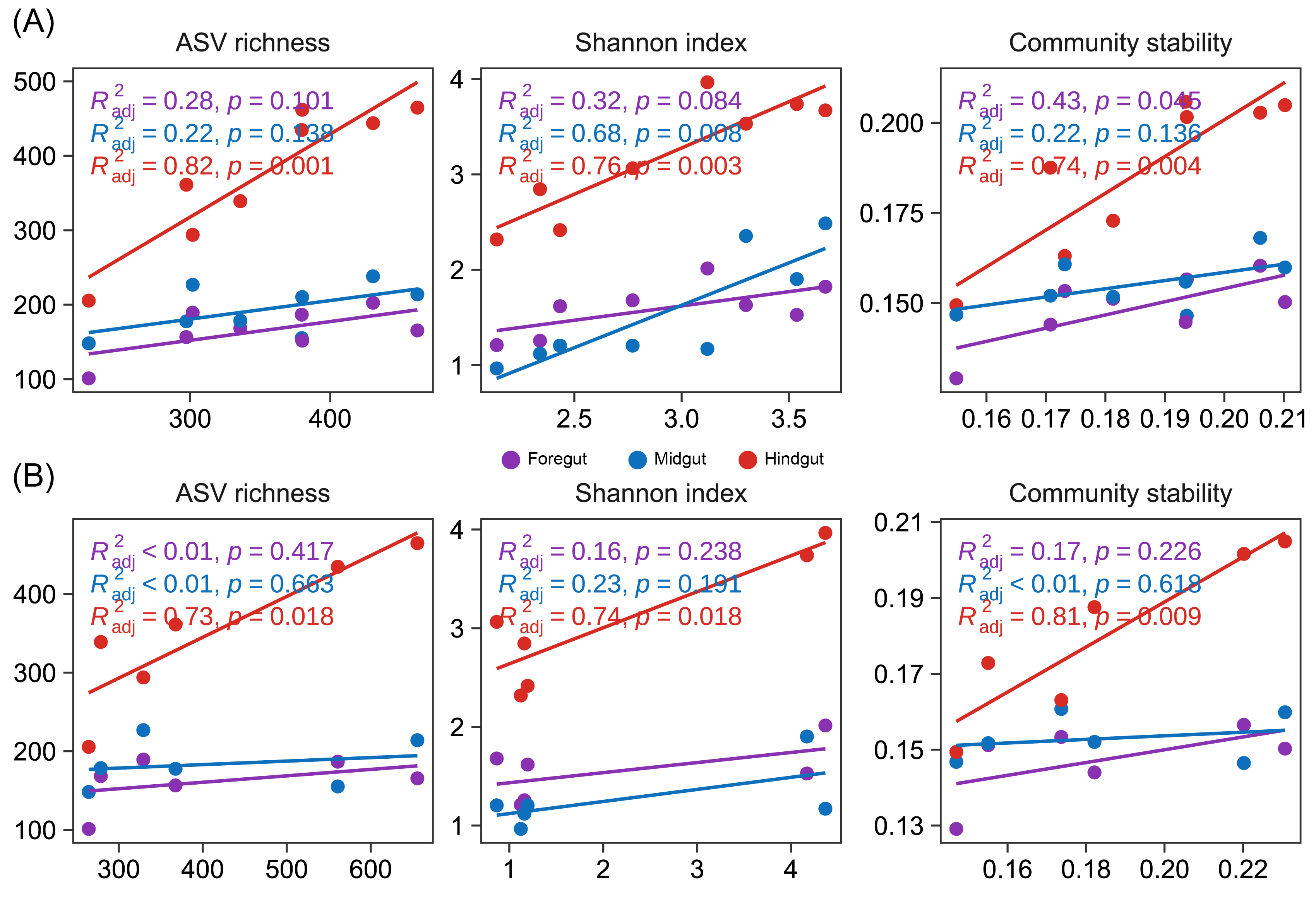


**Figure S4** Linear relationships of α-diversity indices and community stability of bacterial communities between each gut segment and mixed gut (A) or whole gut (B). The adjusted *R*^2^ and *p* values from linear regressions are shown. The color of font and point represent each gut segment, with foregut (purple), midgut (blue), and hindgut (red).


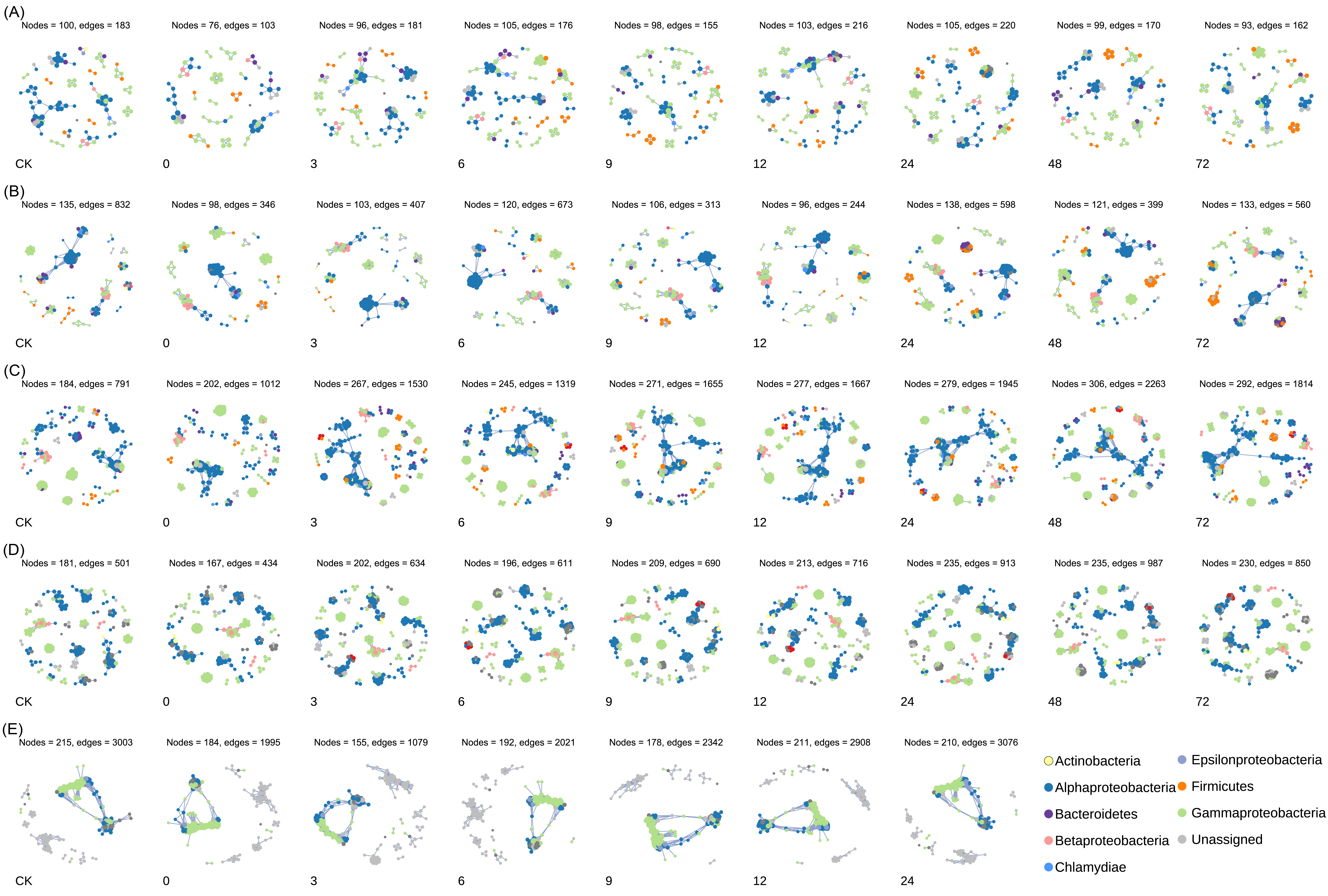


**Figure S5** Co-occurrence networks of bacterial communities in different gut segments. (A) Foregut. (B) Midgut. (C) Hindgut. (D) Mixed gut. (E) Whole gut. The node color represents various phylogenetic phyla. Only robust correlations in which |*r*| (Pearson’s correlation coefficient) was > 0.75 and *p* value was < 0.001 were visualized.


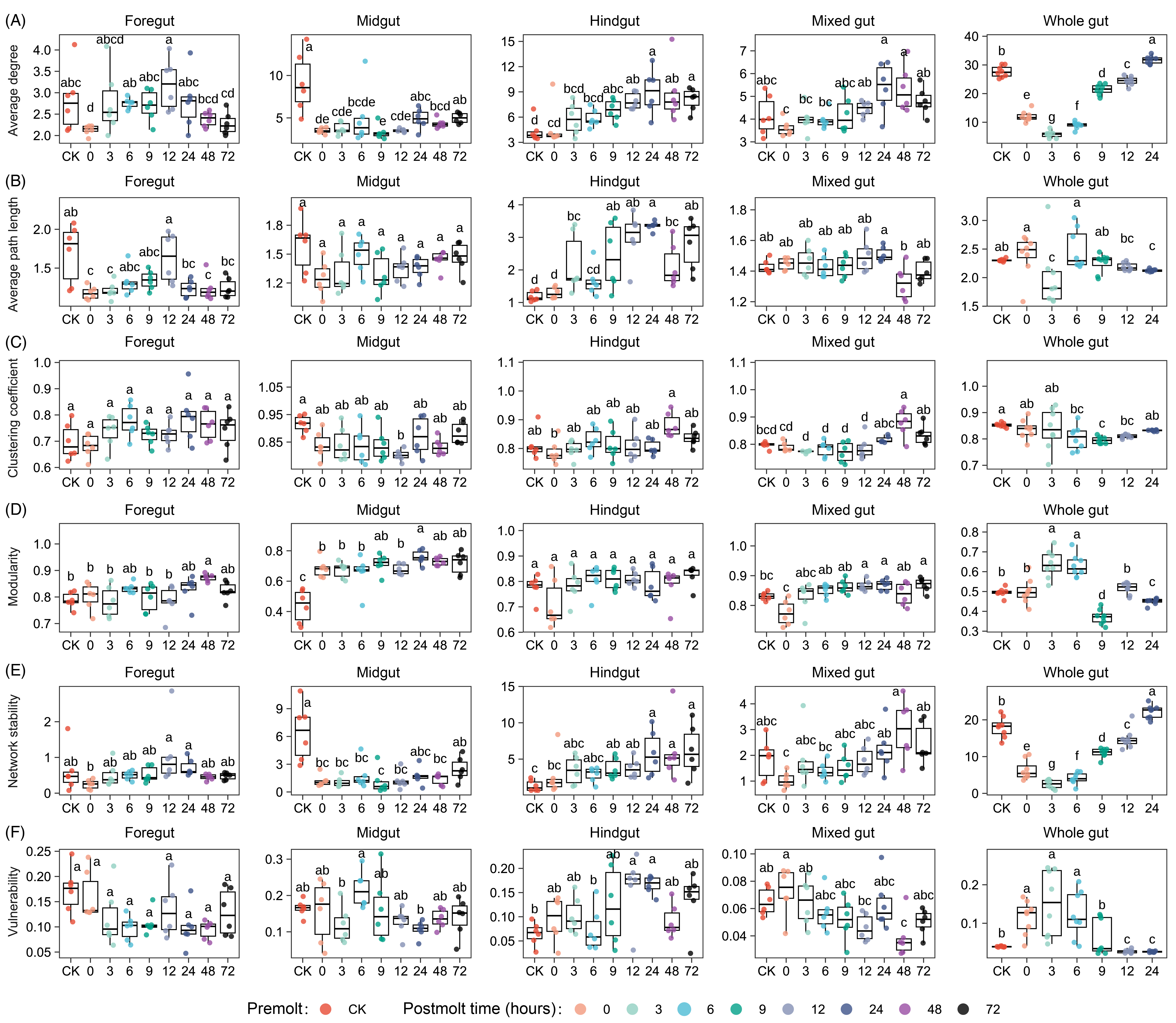


**Figure S6** Temporal changes in network topological attributions. (A) Average degree. (B) Average path length. (C) Clustering coefficient. (D) Modularity. (E) Network stability. (F) Vulnerability. Different lowercase letters indicate significant differences between groups (*p* < 0.05), as determined by Kruskal-Wallis test and Benjamini & Hochberg *p*-value correction.


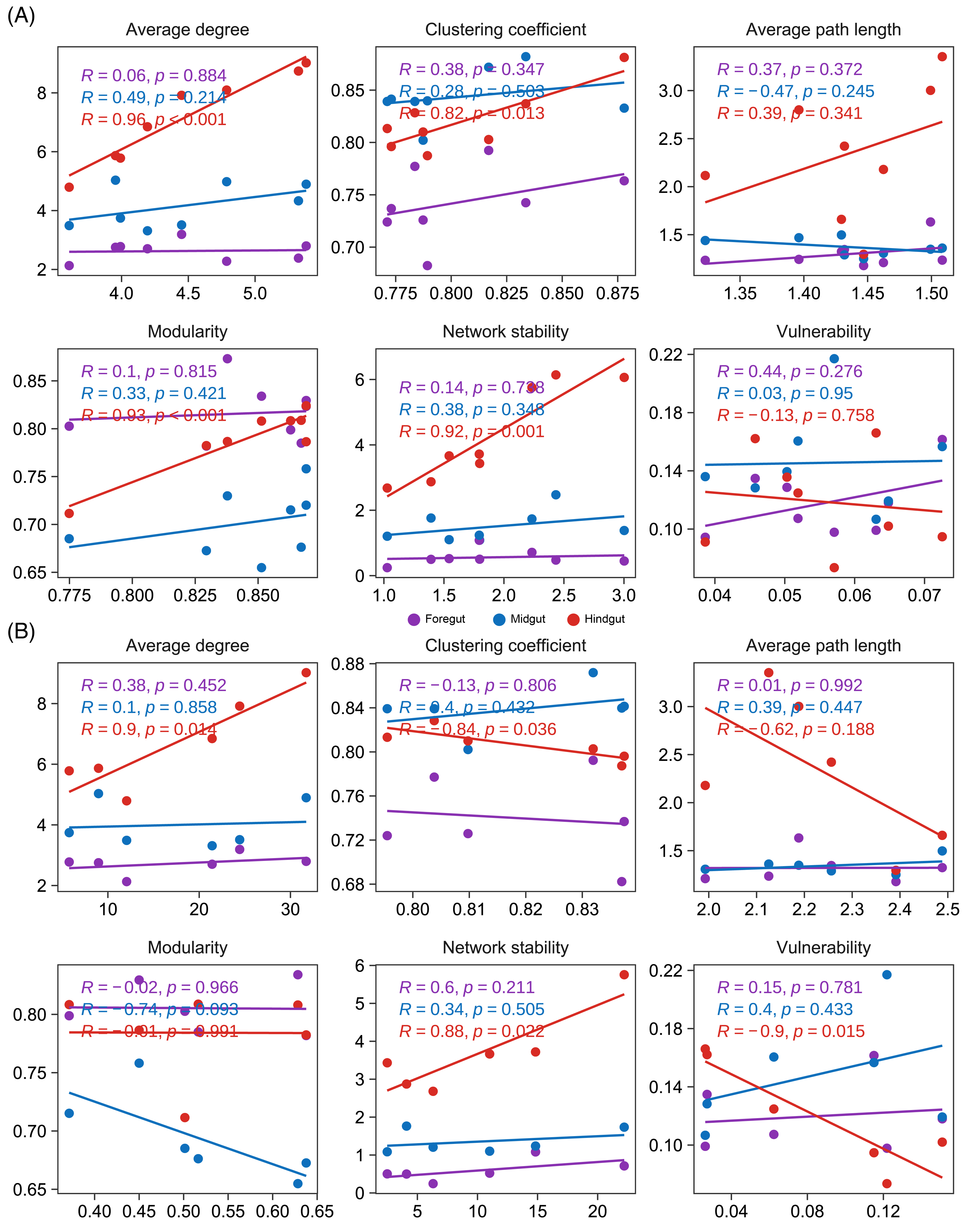


**Figure S7** Linear relationships of network topological attributions between each gut segment and mixed gut (A) or whole gut (B). The adjusted *R*^2^ and *p* values from linear regressions are shown. The color of font and point represent each gut segment, with foregut (purple), midgut (blue), and hindgut (red).


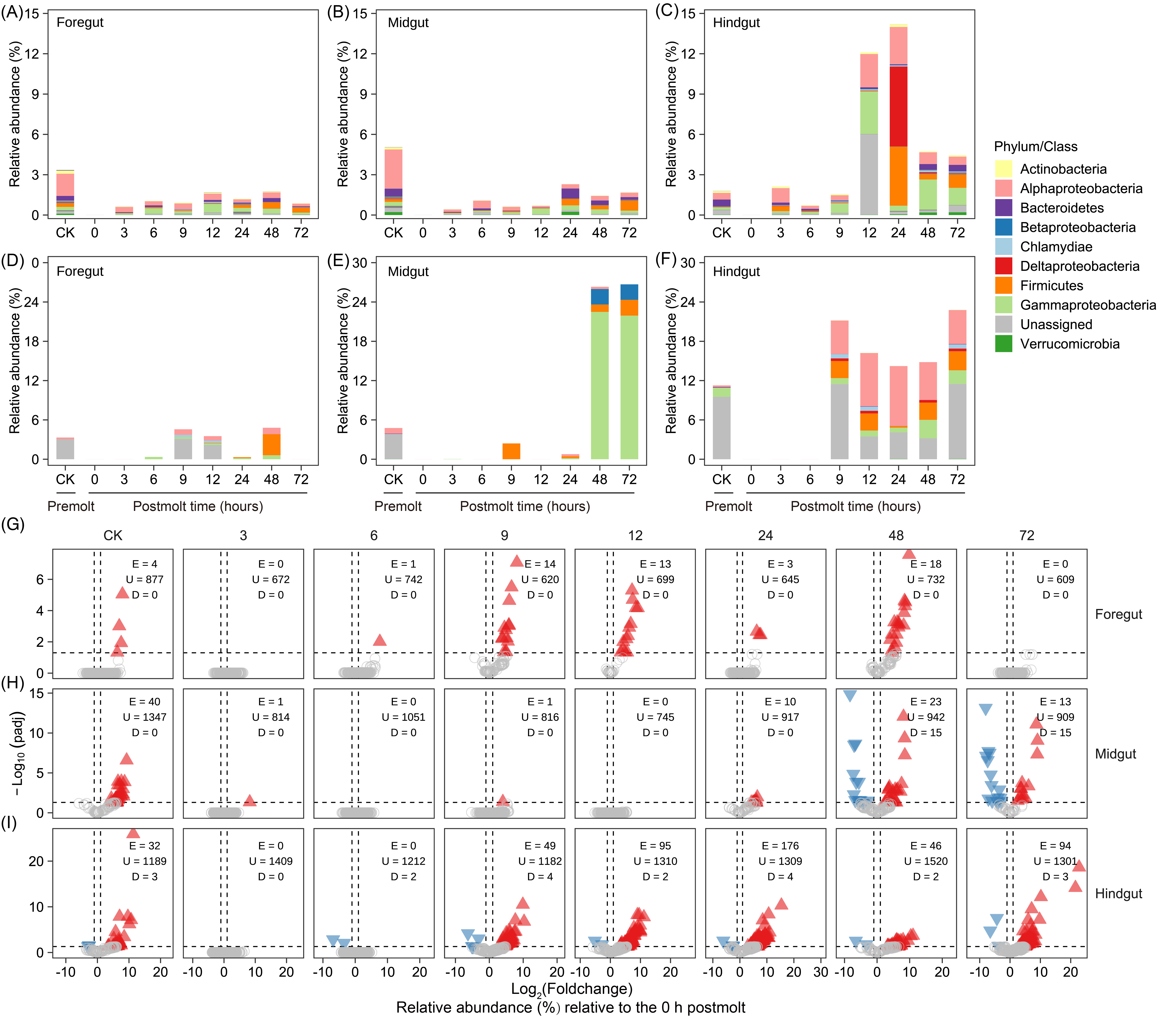


**Figure S8** Temporal changes in the emerged (A–C) and enriched bacterial taxa (D–I) of different gut segments. E refers to enriched ASVs, U refers to unchanged ASVs, and D refers to depleted ASVs. Compared to 0 h postmolt, red triangles represent the enriched ASVs with log_2_ FoldChange >1 and -log_10_ padj >1.3; blue inverted triangles represent the depleted ASVs with log_2_ FoldChange <1 and -log_10_ padj >1.3; gray points represent unchanged ASVs. The two vertical lines correspond to 1.0-fold up and down and the horizontal line represents an adjusted p value equal to 0.05.


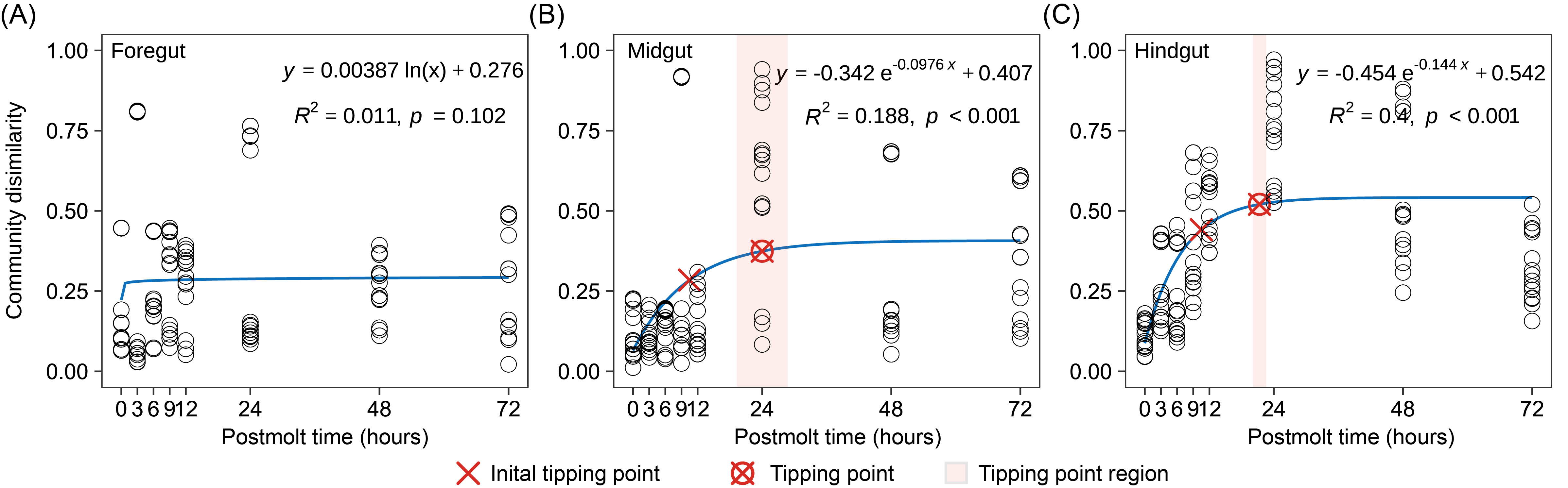


**Figure S9** Tipping points in the changes of bacterial community dissimilarity. (A) Foregut. (B) Midgut. (C) Hindgut. The adjusted *R*^2^ and *p* values from the corresponding regressions are shown. The shapes represent the initial tipping point (cross), tipping point (dot with cross), and tipping point region (square).


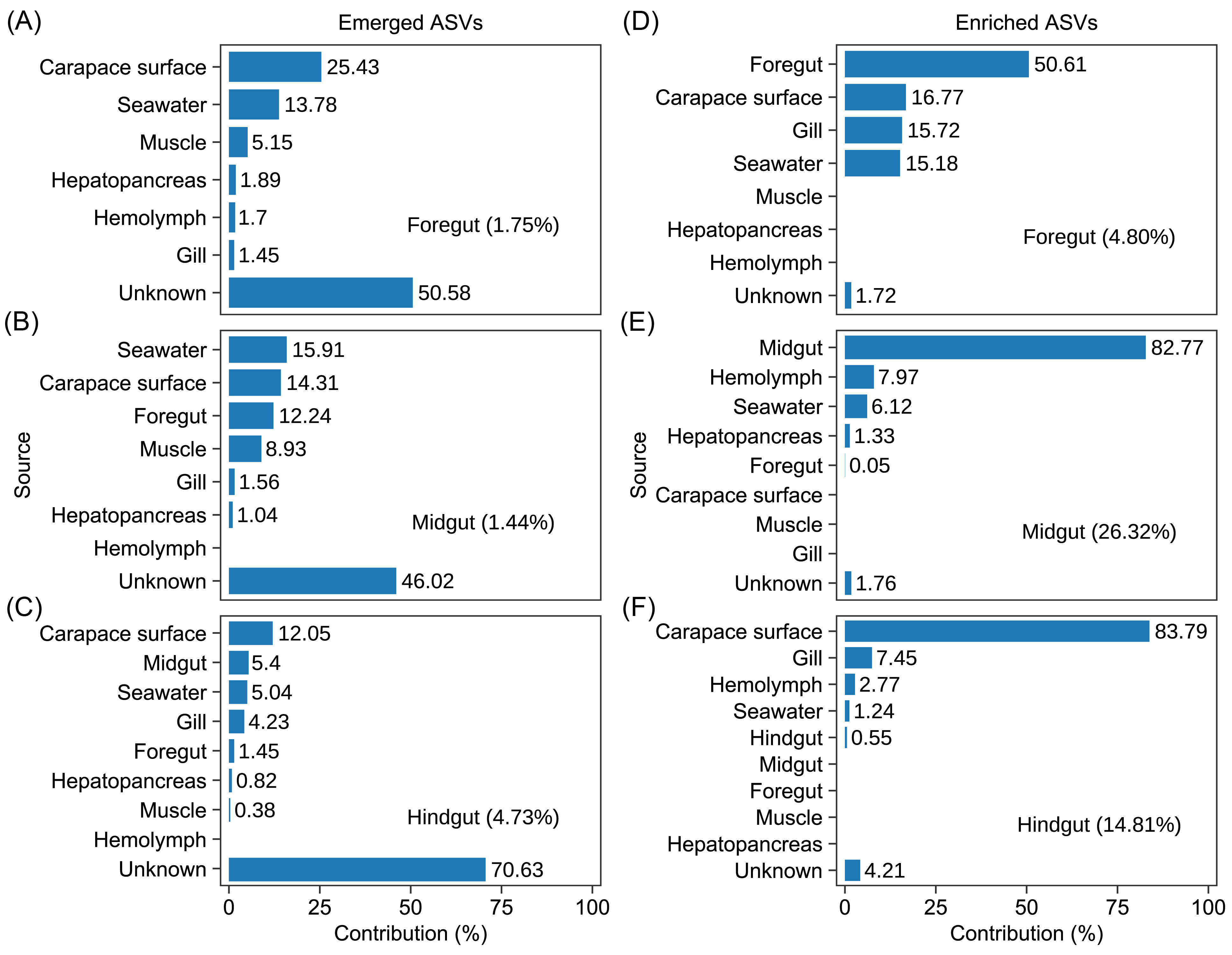


**Figure S10** The potential sources of the emerged (A–C) and enriched bacterial taxa (D–F) at 48 h postmolt. The length of bar with number indicates the source contribution. The number in the lower left represents the relative abundance of emerged or enriched ASVs in each gut segment.


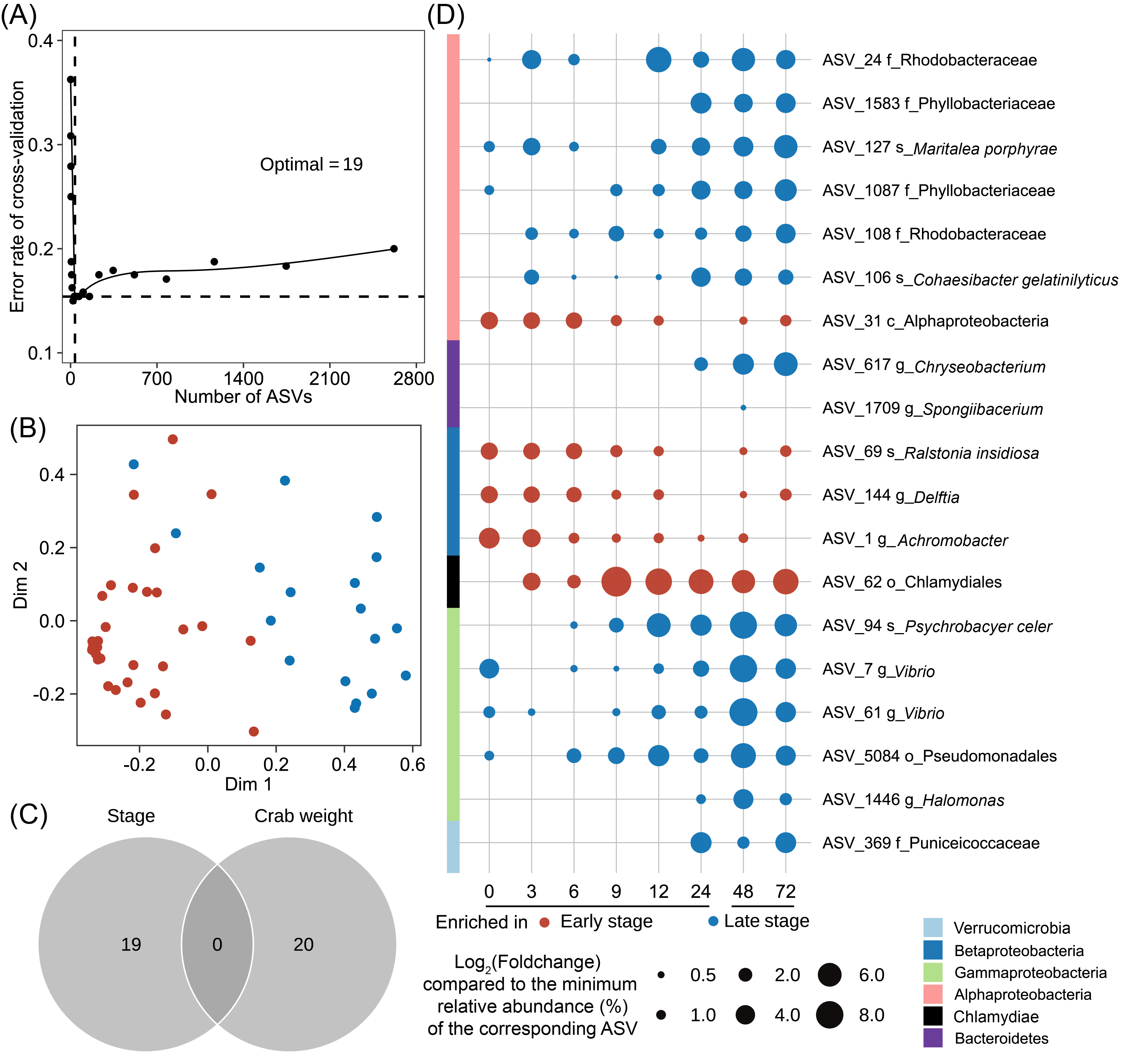


**Figure S11** Bacterial taxa of the hindgut that enriched at the early and late stages during postmolt. (A) Ten-fold cross-validation error as a function of the number of input bacterial taxa used to differentiate early and late stages in order of variable importance. (B) Nonmetric multidimensional scaling (NMDS) plot based on Bray-Curtis dissimilarity visualizing compositional variations of 19 bacterial taxa at the early (red) and late (blue) stages. (C) Venn plot depicting the intersection of bacterial taxa related to stages and crab weight. (D) Changes in the relative abundances of 19 bacterial taxa at the early and late stages. The size of each point represents the fold change compared to the minimum value within each bacterial taxon. The color of the dot represents each bacterial taxa enriched at the early (red) or late (blue) stage. The colors of the bar on the left mean different phyla.


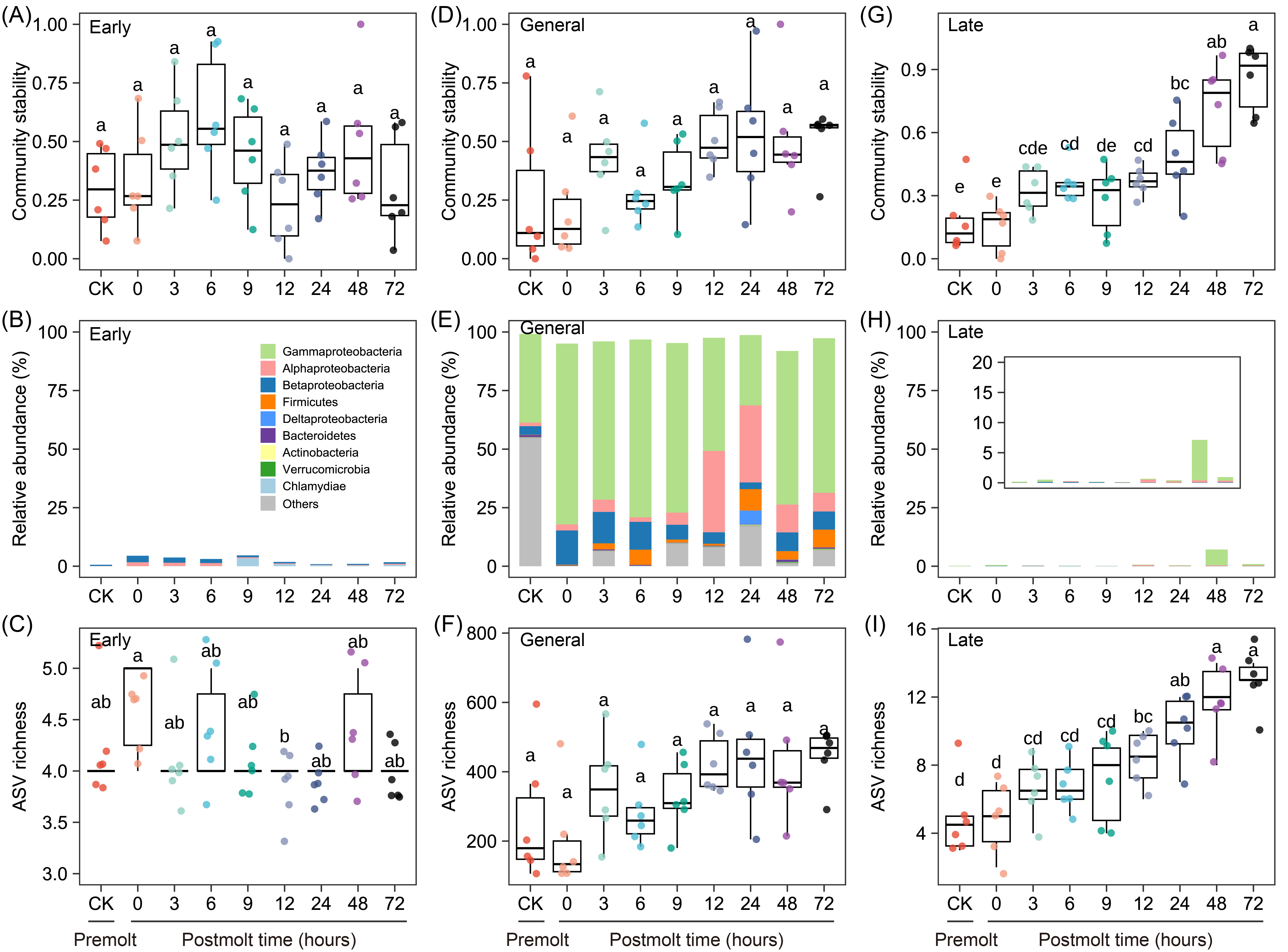


**Figure S12** Community stability, relative abundances, and ASV richness of early (A–C), general (D–F), and late ASVs (G–I) of hindgut bacteria. Early and late ASVs serve as indicators of postmolt early and late stages, respectively. General ASVs represent species that lack distinctiveness in differentiating between the early and late stages. Different lowercase letters indicate significant differences between groups (*p* < 0.05), as determined by Kruskal-Wallis test and Benjamini & Hochberg *p*-value correction.


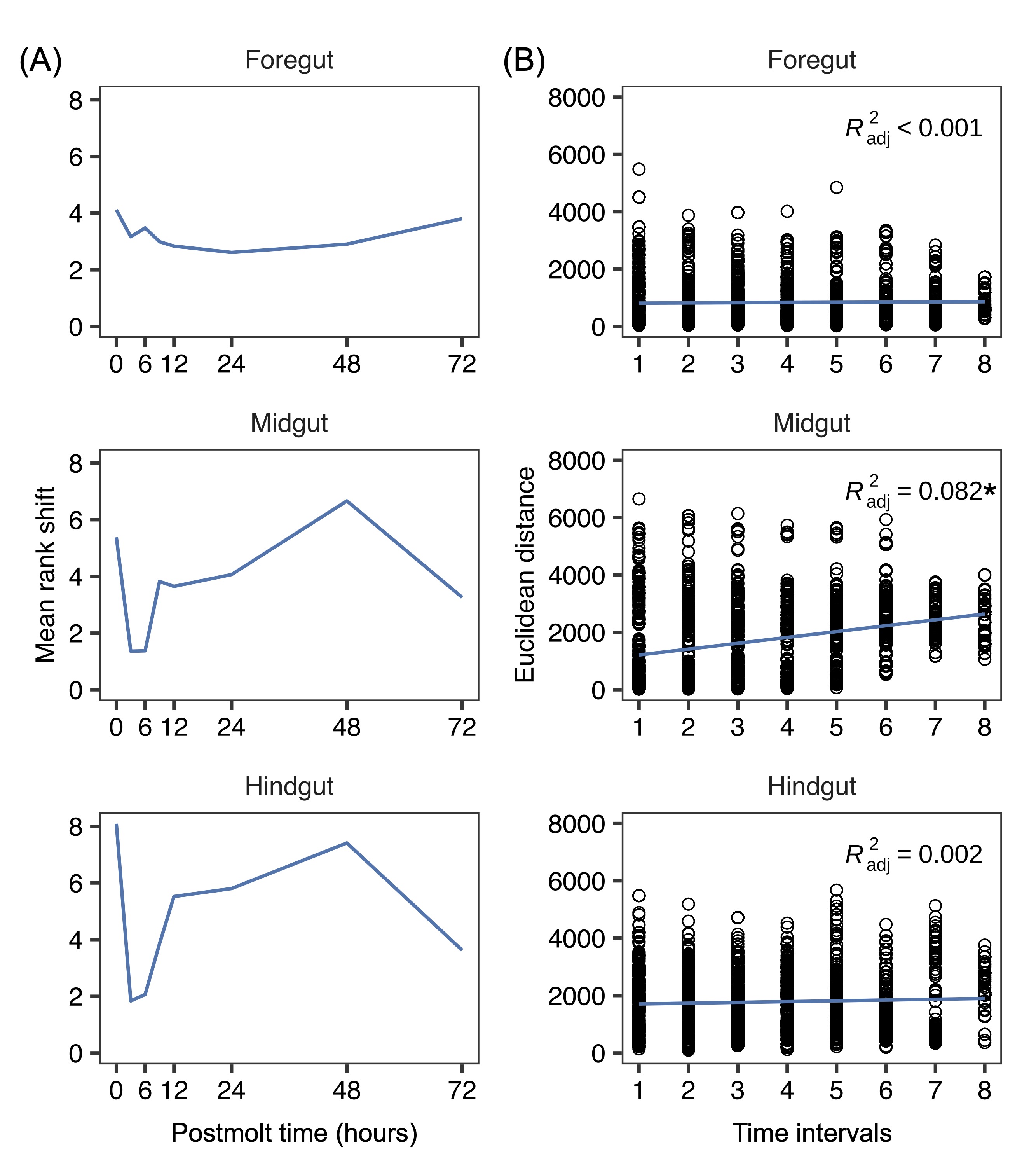


**Figure S13** Bacterial function changes in different gut segments over postmolt time. (A) Mean rank shift. The value was calculated as the average difference in species’ ranks between consecutive time periods, among species that are present across the entire time series. (B) Dissimilarity change in time intervals. Differences in species composition are characterized by Euclidean distances, calculated for pairwise communities across the entire time series. The adjusted *R*^2^ value and *p* value from linear regressions are shown. *, *p* < 0.05.

**Supplemental Methods**

**Experimental design and sample collection**

Two batches of experiments were conducted in this study. The first batch was conducted in July 2021, a total of 276 healthy juvenile crabs with complete appendages (≈ 30 g) were obtained at local aquatic farm in Ningbo, China. Crabs were individually kept in a plastic basket (30 × 19 × 15 cm) covered with transparent organic glass to avoid cannibalism and be easy to observe. Crabs were randomly distributed to 20 rearing tanks (1.5 × 1.0 × 0.6 m) with 300 L of natural seawater per each. During the culturing period, crabs were fed daily with fresh clam (*Ruditapes philippinarum*) meat. The rearing seawater was constantly aerated and maintained under the following conditions: water temperature 28-30 °C, pH 8.0-8.1, salinity 22-25, and dissolved oxygen 5-6 mg/L. After the removal of crab feces and residual feeds, partial seawater (30%) was exchanged once daily.

To accurately determine the postmolt time, surveillance cameras were installed above the rearing tanks. The detailed sampling strategy and timeline are depicted in Graphical abstract. Briefly, nine experimental groups were set up including hard-shell crabs as the control group (CK), soft-shell crabs at 0, 3, 6, 9, 12, 24, 48, and 72 h postmolt (marked as 0, 3, 6, 9, 12, 24, 48, and 72). The samples of gill, hemolymph, muscle, hepatopancreas, foregut, midgut, and hindgut of each crab were collected. The carapace surface was washed with 600 mL of sterilized seawater for 20-30 s. The consulted solution was then filtered through a 0.2-μm sterile polycarbonate membrane (Millipore, USA) for carapace surface bacteria. At the same time, 600 mL rearing seawater was filtered through a 0.2-μm sterile polycarbonate membrane after being pre-filtered with 100-μm sterilized nylon mesh. In this study, whole gut samples were set for eight replications and other samples were set for six replications. Each gut sample was obtained from the gut of each crab. Therefore, six replications were used for each gut segment.

The second batch of experiments was conducted in July 2022. A total of healthy 124 juvenile crabs (≈ 50 g) were purchased at the same aquatic farm and reared in the same way. The whole gut of hard-shell crabs and soft-shell crabs at 0, 3, 6, 9, 12, and 24 h postmolt were collected. Thus, a total of 218 gut samples, 48 other tissue samples at 6 and 24 h postmolt, 12 carapace surface samples, and 12 water samples were collected and stored at -80 °C until further microbiome and metagenomics analysis.

**DNA extraction, 16S rRNA amplicon sequencing**

Total genomic DNA was extracted from the samples using the Power Soil® DNA Kit (MOBIO, USA). The extracted DNA was evaluated for integrity and purity through 1% agarose gel electrophoresis. The DNA concentration and purity were determined using the NanoDrop One spectrophotometer (Thermo Fisher Scientific, MA). To analyze bacterial community, a specific region of the 16S rRNA gene was amplified using the 338F/806R primer pairs (5′-ACTCCTACGGGAGGCAGCAG-3′ and 5′-GGACTACHVGGGTWTCTAAT-3′) [1]. Sequencing libraries were prepared using the NEBNext® UltraTM DNA Library Prep Kit for Illumina® (New England Biolabs, MA), following the manufacturer's instructions. Index codes were incorporated, and the library quality was assessed using the Qubit@ 2.0 Fluorometer (Thermo Fisher Scientific, MA) and Agilent Bioanalyzer 2100 system (Agilent Technologies, Waldbron, Germany). Finally, the library was sequenced on an Illumina Novaseq platform (San Diego, CA), generating 250 bp paired-end reads. The acquired sequences were filtered for quality control using the USEARCH tool [2]. After filtering, clean raw data were merged into the bacterial tags and further clustered into amplicon sequence variants (ASVs) with 100% sequence similarity. Based on the RDP database, the bacterial ASVs were taxonomically classified by the RDP classifier. The sequence of each sample was normalized to minimum sequence depth (34,848) for further fair analysis. According to Kyoto Encyclopedia of Genes and Genomes (KEGG) orthologs, phylogenetic investigation of the bacterial community by reconstruction of observed states (PICRUSt2, v2.1.0-b) pipeline in the server was used to predict functional potentials of the gut bacterial community [3].

**Bacterial community analysis**

The stability of the bacterial community was calculated based on the ratio of the mean to the standard deviation per sample. The α- and β-diversities of bacterial communities were analyzed using the vegan and Rtsne packages, respectively. The difference of each α-diversity index and community stability was checked using the Kruskal-Wallis test and BH *p*-value correction. Relative to the ASVs in the gut at 0 h postmolt, the emerged and significantly enriched ASVs in the gut at premolt and at other time points postmolt were assessed using DESeq2, with Log_2_ FoldChange > 1 and BH adjusted *p* < 0.05 as criteria. To further identify distinctive bacterial ASVs between groups, a random forest classification model and cross-validation were conducted using the Randomforest package. Fast expectation-maximization microbial source tracking (FEAST) method was used to reveal the sources of the emerged and enriched bacteria. Linear regression was used to test the correlations among the indices of bacterial data.

The pairwise interactions among bacterial ASVs were calculated using WGCNA package. An interaction was robust when Pearson’s correlation coefficient > 0.75 and BH adjusted *p*-value < 0.001. The acquired adjacency matrix was generated into a co-occurrence network using the igraph package. The topological features of each network were calculated using the igraph package, including nodes, clustering coefficient, average path length, average degree, and modularity. To evaluate the stability of each network, the robustness index was quantified by calculating the proportion of taxa that remained when randomly removing 50% of taxa from each network [4]. Network visualization was performed using the ggraph package.

**Metagenomics sequencing**

The same DNA templates from the midgut samples at premolt as well as at 0, 12, and 48 postmolt were used for shotgun metagenomic sequencing. The corresponding libraries were sequenced on an Illumina NovaSeq 6000 platform, generating 150 bp paired-end reads. After the removal of adaptor sequences and low-quality reads, the high-quality reads were further assembled into contigs using MEGAHIT (Version v1.0.6). Open reading frames in contigs were predicted using MetaGeneMark software (Version v1.0.6). The predicted genes from each sample were merged and clustered using CD-Hit software (Version: 4.7) based on the criteria of the identity of > 95% and coverage of > 90% to remove redundant genes. A non-redundant gene matrix was constructed using the Salmon software. The functions of genes were annotated based on the KEGG database by Diamond software (https://github.com/bbuchfink/diamond/).

**Functional data analysis**

Measurements of the relative change and the differences of functional composition between consecutive periods using the codyn package. Function stability was calculated based on the ratio of the mean of functional abundance to the standard deviation per sample, and the multifunctionality index was represented by averaging the standardized scores. Kruskal-Wallis test and BH *p*-value correction were used to analyze the differences in each functional profile (KEGG level 3), functional stability, and multifunctionality. In addition, the main bacterial drivers and their contributions to functional stability and multifunctionality were identified using a Random Forest regression model. The significance of the model was assessed with 5000 permutations of the response variable using the A3 package. The correlations between the species and functions were tested using the psych package. Finally, the FEAST method was used to reveal the source of the species that which significantly related to functions.

**Supplemental References**

1. Castrillo, Gabriel, Paulo José Pereira Lima Teixeira, Sur Herrera Paredes, Theresa F. Law, Laura de Lorenzo, Meghan E. Feltcher, Omri M. Finkel, *et al.* 2017. “Root microbiota drive direct integration of phosphate stress and immunity.” *Nature* 543: 513–518. https://doi.org/10.1038/nature21417

2. Robert C. Edgar. 2010. “Search and clustering orders of magnitude faster than BLAST.” *Bioinformatics* 26(19): 2460–2461. https://doi.org/10.1093/bioinformatics/btq461

3. Douglas M. Gavin, Vincent J. Maffei, Jesse R. Zaneveld, Svetlana N. Yurgel, James R. Brown, Christopher M. Taylor, Curtis Huttenhower & Morgan G. I. Langille, 2020. “PICRUSt2 for prediction of metagenome functions.” *Nature Biotechnology* 38(6): 685–688. https://doi.org/10.1038/s41587-020-0548-6

4. Yuan M. Mengting, Xue Guo, Linwei Wu, Ya Zhang, Naijia Xiao, Daliang Ning, Zhou Shi, et al. 2021. “Climate warming enhances microbial network complexity and stability.” *Nature Climate Change* 11: 343–348. https://doi.org/10.1038/s41558-021-00989-9
